# Silent clusters – speak up!

**DOI:** 10.1111/1751-7915.12181

**Published:** 2014-12-27

**Authors:** Lone Gram

**Affiliations:** Department of Systems Biology, Technical University of DenmarkMatematiktorvet bldg. 301, DK-2800, Kgs. Lyngby, Denmark

Microorganisms have provided mankind with a multitude of useful compounds ranging from industrial enzymes to anti-cancer compounds and antibiotics. With the aging population, our need for treatment principles against cancer, Alzheimer's disease and metabolic disorders is increasing. Also, the occurrence and spread of antibiotic resistance in pathogenic microorganisms has become one of our main challenges. For a number of years, it was hoped that our advances in chemical synthesis would provide the desired novelty in terms of scaffolds and targets, however, while there has been success, it has also been realized that the complexity of natural (microbial) products far surpasses anything possible by chemical synthesis. Hence, drug discovery has returned to nature, especially microorganisms, as a source of novel chemistry. Recently, genome sequencing and mining has demonstrated that bioactive compounds hitherto believed to be produced by a eukaryotic organism (a marine sponge) were indeed synthesized by one of the dominant (uncultured) prokaryotes, ‘*Entotheonella*’ further underlining the vast bioactive underestimated potential in microorganisms (Wilson *et al*., 2014).

Classical bioassay guided fractionation has been used for decades as the major bioprospecting path, however, this is often a laborious process, and rediscovery of known compounds is a major challenge despite developments in de-replication strategies. The rapidly developing sequencing techniques are introducing sequencing of microbial genomes in almost any microbial research environment, and hence so-called genome mining has become a useful tool in discovery of novel microbial products (Jensen *et al*., 2014). Despite the complexity of the bioactive secondary metabolites produced by microorganisms, several pathways are conserved at the modular level. This has allowed the development of software, such as antiSMASH or NaPDoS, which can identify characteristic structures of polyketide and non-ribosomal peptide synthetases (Weber 2014). Such genome mining processes have revealed that the bioactive potential in numerous microorganisms is likely much higher than previously discovered by bioassay-guided fractionation. This has been demonstrated for well-known producers of antibiotics, in *Actinobacteria* and fungi. However, it is also true for several groups of Gram-negative prokaryotes (Still *et al*., 2014) that hitherto have not been thought of as useful producers of bioactive compounds. For instance in *Pseudoalteromonas luteoviolaceae*, 3–4 bioactive compounds have been isolated (violacein, indolmycin, pentabromopseudilin), however, genome mining reveals approximately 15 gene clusters likely encoding for pathways likely producing bioactives (H. Machado, unpublished).

It is believed that the reason for the ‘non-discovery’ of these potential novel compounds is largely because the genes are not expressed during normal laboratory cultivation; so-called silent gene clusters. Hence, the activation or use of such silent clusters is becoming a hot research topic. How to unlock this potential? How to make the silent clusters ‘speak up'? Several paths are being pursued. One is based on synthetic biology with the approach of cloning the clusters and expressing in heterologous hosts. There are examples of success from this approach (Ross *et al*., 2014), and more are likely to come. However, there are also several challenges. The gene clusters encoding putative bioactive compounds are typically large (10–100 kb) and often consists of many genes. Hence, the cloning and expression is significantly more challenging than cloning of a single gene and expressing its product. Also, the clusters/genes may fail to express in a heterologous host due to lack of signals naturally present in the native producer.

Another path involves the modification of gene expression in the natural producer organism in a general sense. In fungi, inhibitors of histone deacetylation can induce secondary metabolism by increasing promoter accessibility due to changes in chromatin structure. Analogously, expression of otherwise silent gene clusters in *Streptomyes coelicolor* has been demonstrated using histone deacetylation inhibitors as elicitors (Moore *et al*., 2012). Japanese researchers have successfully used genetic modification of the producer microorganism to elicit bioactive compound production. Approaches include ‘ribosome engineering’ as well as overexpression of regulator proteins (Ochi and Hosaka, 2013).

A broad range of approaches all fall under the same overall strategy, involving variation of growth conditions of the potential producing organism with subsequent chemical analyses (LC-MS, MS/MS) and bioassays. A vast amount of studies have demonstrated that the production of secondary metabolites in microorganisms is dependent on growth substrate and environment. Stress signals, such as antibiotics (Seyedsayamdost, 2014), ppGpp, nutrient depletion or physical stress can induce silent clusters and production of bioactives. Also, several groups are working on culture approaches where the conditions are mimicking the natural chemical and/or biological environment of the potential producing microorganisms (Schroeckh *et al*., 2009; Lincke *et al*., 2010). This also covers switching from test tubes and flasks to using biofilm mode of growth, co-cultivation systems (König *et al*., 2013) and natural co-occurring nutrients. Especially, the production of novel compounds as a result of co-culturing has been beautifully demonstrated by use of direct chemical probing (nanospray desorption electrospray ionization and microbial matrix-assisted laser desorption ionization-time of flight imaging mass spectrometry) (Traxler *et al*., 2013). We demonstrated (Wietz *et al*., 2011) that culturing marine bacteria on chitin, the dominant organic polymer in marine systems, as opposed to glucose dramatically altered the secondary metabolism, and the bacterium focused its production on bioactive compounds. In a simple agar screen for antibiotic activity, we recently found that if the producer organisms were grown on chitin, the number of isolates with antibacterial activity tripled (S. Giubergia, unpublished).

Likely, the future will see a combination of several approaches and technologies. Aligning manipulations of culture conditions (natural substrates, associate microbiota) with chemical (metabolomic molecular networking), genome and metagenome-based analyses is indeed likely to enhance our bio-discovery hit rate and provide novel understanding of the ecology and natural interactions of microorganisms.

## Conflict of interest

Authors have no conflict of interest to declare.
